# The impact of lactoferrin with different levels of metal saturation on the intestinal epithelial barrier function and mucosal inflammation

**DOI:** 10.1007/s10534-016-9973-x

**Published:** 2016-10-18

**Authors:** Grzegorz Majka, Grażyna Więcek, Małgorzata Śróttek, Klaudyna Śpiewak, Małgorzata Brindell, Joanna Koziel, Janusz Marcinkiewicz, Magdalena Strus

**Affiliations:** 1 Chair of Microbiology, Jagiellonian University Medical College, Czysta 18, 31-121 Kraków, Poland; 2 Chair of Immunology, Jagiellonian University Medical College, Czysta 18, 31-121 Kraków, Poland; 3 Department of Inorganic Chemistry, Faculty of Chemistry, Jagiellonian University, Ingardena 3, 30-060 Kraków, Poland; 4 Department of Microbiology, Faculty of Biochemistry, Biophysics and Biotechnology, Jagiellonian University, Gronostajowa 7, 30-387 Kraków, Poland

**Keywords:** Lactoferrin, Sepsis, Bacterial translocation, Iron, Manganese

## Abstract

Translocation of bacteria, primarily Gram-negative pathogenic flora, from the intestinal lumen into the circulatory system leads to sepsis. In newborns, and especially very low birth weight infants, sepsis is a major cause of morbidity and mortality. The results of recently conducted clinical trials suggest that lactoferrin, an iron-binding protein that is abundant in mammalian colostrum and milk, may be an effective agent in preventing sepsis in newborns. However, despite numerous basic studies on lactoferrin, very little is known about how metal saturation of this protein affects a host’s health. Therefore, the main objective of this study was to elucidate how iron-depleted, iron-saturated, and manganese-saturated forms of lactoferrin regulate intestinal barrier function via interactions with epithelial cells and macrophages. For these studies, a human intestinal epithelial cell line, Caco-2, was used. In this model, none of the tested lactoferrin forms induced higher levels of apoptosis or necrosis. There was also no change in the production of tight junction proteins regardless of lactoferrin metal saturation status. None of the tested forms induced a pro-inflammatory response in Caco-2 cells or in macrophages either. However, the various lactoferrin forms did effectively inhibit the pro-inflammatory response in macrophages that were activated with lipopolysaccharide with the most potent effect observed for apolactoferrin. Lactoferrin that was not bound to its cognate receptor was able to bind and neutralize lipopolysaccharide. Lactoferrin was also able to neutralize microbial-derived antigens, thereby potentially reducing their pro-inflammatory effect. Therefore, we hypothesize that lactoferrin supplementation is a relevant strategy for preventing sepsis.

## Introduction

Despite advances in contemporary neonatology and superior hygienic conditions, infections continue to affect newborns. Neonates with a very low birth weight (VLBW; e.g., <1500 g) are at particular risk of bloodstream infections which are associated with high rates of morbidity and mortality (Lawn et al. 2006; Shane and Stoll 2014). The infections may originate from a hospital environment or may arise from the translocation of microorganisms from the intestinal lumen to the peripheral blood supply. Disruption of mucosal barrier function can occur following a compromise of intestinal epithelial cell integrity, in the presence of immune cells of the mucosa-associated lymphoid tissue (MALT) system, and during the infiltration of inflammatory cells which facilitates the translocation of microorganisms (Deitch 2012; Sherman 2010).

In full-term newborns, microbiota originating from their mothers’ urogenital and gastrointestinal tract undergo natural colonization. However, this colonization is disturbed in VLBW infants with a lack of breastfeeding and during antibiotic treatment. As a result, a decrease in the population numbers of beneficial bacteria, such as *Bifidobacterium* and *Lactobacillus* genera, occurs in a newborn’s gastrointestinal tract (Gritz and Bhandari 2015). In addition, gut microbiota of VLBW neonates become populated with potentially pathogenic species of *Enterobacteriaceae* (e.g., *Escherichia coli*, *Klebsiella pneumoniae*) and *Staphylococcaceae* (e.g., *Staphylococcus aureus*) families. Intestinal dysbiosis, as well as immaturity of the gut and mucosal immunity, then contribute to a loss of epithelium integrity and the translocation of microorganisms from the lumen to the peripheral blood supply.

Lactoferrin (Lf) is an 80-kDa glycoprotein that belongs to the transferrin family and is abundant in the colostrum and milk of mammals. The concentration of Lf in colostrum is 5–13 g/l and it is 1.5–4.5 g/l in milk (Hamosh 1998; Ronayne de Ferrer et al. 2000). Therefore, it is plausible that this protein may play an important role during neonatal development when the daily supply of Lf in an infant ranges from 0.4 g to 1.2 g/kg body weight (Sánchez et al. 1992).

Iron is an element that is crucial for the growth of most microorganisms which have developed efficient mechanisms for uptake of this metal. Lf is capable of binding two ferric ions with very high affinity, and it is this property that is typically associated with the antimicrobial activity of Lf (Aisen and Leibman 1972; Baker et al. 1994; Paulsson et al. 1993). Two additional forms of Lf include a metal-free form, known as apolactoferrin (apoLf), and an iron-saturated form named hololactoferrin (holoLf). Lf can also bind other metals in its two binding sites. Recently, we described the synthesis and prebiotic properties of a manganese-saturated form of Lf, MnLf, towards several *Lactobacillus* strains (manuscript in review).

However, while many publications have described the mechanism of metal binding by Lf, the impact of lactoferrin metal saturation status has been not explored enough in the context of bacterial translocation. Firstly, apolactoferrin used in previous studies was estimated to be 10 % iron-saturated which is saturation commonly found in the native protein preparations (Goldoni et al. 2000; Marchetti et al. 1998; Superti et al. 2001; Valenti et al. 1999). Our aim was to reach minimal possible iron saturation and compare such apolactoferrin activity with that of a native protein as well as forms with significant saturation with iron and manganese. Also, we focused on evaluating importance of iron saturation status of lactoferrin for bacterial translocation in the context of neonatal sepsis. Reports on manganese-saturated lactoferrin have been mostly focused on its potential to mitigate intracellular infections with both viruses (HSV1, SA-11) (Marchetti et al. 1998; Superti et al. 2001) and bacteria such as *Listeria monocytogenes* and *Legionella pneumophila* (Goldoni et al. 2000; Valenti et al. 1999). To our best knowledge, lactoferrin saturated with manganese have not been reported in studies related to maintaining intestinal epithelium cells integrity and limiting inflammatory response to microbial antigens in the gut.

The presence of Lf at high concentrations in human colostrum (~7 g/l) and milk (1–2 g/l) suggests the importance of this protein in maintaining homeostasis in the neonatal gut niche. Human Lf is known to bind a specific receptor (LfR) that is expressed by intestinal epithelium cells and may be internalized upon binding (Suzuki et al. 2001). At high concentrations (e.g., >100 µg/ml), both bovine and human Lf induces the proliferation of enterocytes, whereas lower concentrations of Lf (e.g., <100 µg/ml) promote the differentiation of enterocytes (Blais et al. 2014; Buccigrossi et al. 2007). In an in vitro model of epithelial cells, human Lf has been shown to increase the integrity of Caco-2 cells based on measurements of electrical resistance and dextran permeability (Hirotani et al. 2008). However, the impact of Lf on tight junction proteins (e.g., claudin, occludin) has not been described in the literature despite the role of these proteins in maintaining the integrity of the intestinal epithelium. It is also worth mentioning that native bovine Lf has not exhibited toxicity across a wide range of concentrations and does not induce apoptosis or necrosis in epithelial cells in vitro (Ajello et al. 2002).

In addition to antimicrobial activities, Lf may also affect the immune response. It has been hypothesized that Lf plays a role in the innate immune system that provides a first line of defense against infections (Legrand 2011). The latter activity is based on interactions between Lf and bacteria-derived molecules, particularly lipopolysaccharide (LPS). For example, human Lf was shown to bind and neutralize lipid A of LPS, thereby preventing lipid A-mediated stimulation of immune cells (Appelmelk et al. 1994; Legrand et al. 2005). It has also been demonstrated that LPS binding by both human and bovine Lf affects the production of cytokines and reactive oxygen species (Ambruso and Johnston 1981; Baveye et al. 2000; Håversen et al. 2002). However, there are no data available on whether the various forms of Lf differentially affect the immune response.

Bovine Lf is a promising therapeutic agent that has already been tested in a few clinical trials (Manzoni et al. 2009, 2012; Ochoa et al. 2015). In most of these trials, native bovine Lf with an unknown level of iron saturation was used. It is important to note that the metal saturation status of a protein has the potential to affect the conformation and possible interactions of the protein with other molecules. As a result, metal saturation status may alter the biological activity of Lf. Therefore, it is crucial to perform basic studies regarding the mode(s) of action of the various Lf forms in relevant models.

The present study had three aims. The first was to evaluate the biological activities of various bovine Lf forms that differ in their metal saturation levels. Namely, apoLf, holoLf, and MnLf were compared with naturally occurring (native) Lf. A high concentration was used for each of the forms (5 mg/ml) based on the levels of Lf that have been detected in human colostrum and the levels used for diet supplementation of VLBW neonates. We especially focused on the role of the different iron and manganese saturation levels of Lf on the viability and integrity of the intestinal epithelium barrier that is dependent on the presence of tight junctions. Second, we investigated whether metal binding by Lf affects the intestinal epithelium and the ability of immune cells to secrete pro- and anti-inflammatory cytokines. Third, we attempted to confirm whether various Lf forms retain the native ability of Lf to diminish the inflammatory response by interacting with bacterial components such as LPS.

## Materials and methods

### Lf preparations

Bovine Lf was purchased from Friesland Campina, Netherlands. Chemical purity of the native Lf preparation was estimated to be >90 % and its iron saturation ca. 10.2 ± 0.2 %. ApoLf and holoLf were prepared as previously described (Majka et al. 2013) with iron saturation values of 1.2 ± 0.2 and 71.8 ± 6.5 %, respectively. To obtain apoLf, the original Lf preparation was dialysed against 0.1 M citrate buffer (pH 4.0) for 24 h to remove ferric ions. The buffer was then exchanged with deionized water for a dialysis step that was performed over 24 h. HoloLf was prepared by reacting an original Lf preparation with ferric nitrate(V) in the presence of a weak chelating agent, nitrilotriacetic acid (NTA), in a molar ratio of 1:4:4 of Lf:ferric ions:NTA. MnLf (47.1 ± 2.0 % manganese saturation) was prepared using reaction with manganese(II) citrate (prepared by reaction of manganese(II) chloride hydrate and citric acid). Briefly, ferric ions were removed (as described for apoLf) and then the metal-deficient Lf was reacted with 20-fold molar excess of manganese in HEPES buffer (pH 7.5, 100 mM NaCl, 25 mM NaHCO_3_) for 24 h at 37 °C. Both holoLf and MnLf were subjected to further dialysis against deionized water (24 h in room temperature with three changes of water) to remove nonspecifically bound metals. Iron and manganese saturation was estimated using ICP-OES (metal content) and ELISA (Lf concentration) and summarized in Table 1.

**Table 1:** Iron and manganese saturation of bovine lactoferrin preparations

Lf form	Fe (%)	Mn (%)
apoLf	1.2 ± 0.2	Less than 0.5
nLf	10.2 ± 0.2	Less than 0.5
holoLf	71.8 ± 6.5	Less than 0.5
MnLf	Less than 0.5	47.1 ± 2.0

### Cell culture

A. The human intestinal epithelial cell line, Caco-2 (ATCC^®^ HTB-37™), was purchased from American Type Culture Collection (ATCC). Cells were cultured in Minimum Essential Medium (MEM) containing 2 mM l-glutamine (Lonza, Switzerland), 5 % fetal bovine serum (FBS), and antibiotics (penicillin/streptomycin) at 37 °C in a humidified 5 % CO_2_ atmosphere. Cells were passaged every 3–5 days using trypsin.
B. Human monocyte-derived macrophages (hMDMs) were isolated from peripheral blood mononuclear cells donated by healthy donors to the Red Cross (Kraków, Poland). Since the Red Cross deidentifies blood materials, as is appropriate for human subject confidentiality assurances, consent from patients or approval by any institutional review board for use of these blood materials was not necessary. Peripheral blood mononuclear cells (PBMCs) were isolated from human blood using a lymphocyte separation medium density gradient that yielded a fraction that was highly enriched in monocytes (90 % CD14-positive), as described previously (Koziel et al. 2014). These cells were subsequently cultured in RPMI 1640 (Lonza, Switzerland) that was supplemented with 2 mM l-glutamine, 50 μg/ml gentamycin, and 10 % autological human serum. Cells were plated at 3 × 10^6^/well in 24-well plates (Sarstedt, Numbrecht, Germany) in RPMI 1640 supplemented with 2 mM l-glutamine, 50 μg/ml gentamycin, and 10 % autological human serum. After 24 h, nonadherent PBMCs were removed by washing with complete medium, and adherent cells were cultured in this medium for 7 days, with media changed every 2 days up to their differentation into hMDMs.
C. The murine monocyte/macrophage cell line, J774A.1 (ATCC^®^ TIB-67™), was purchased from ATCC. Cells were cultured in High-Glucose Dulbecco’s Modified Eagle’s Medium (DMEM) supplemented with 2 mM l-glutamine, 10 % FBS, and antibiotics (penicillin/streptomycin). The cells were cultured for 5–7 days before being used for experiments.

### Apoptosis/necrosis assay

Briefly, Caco-2 cells were seeded in 24-well plates (5 × 10^5^ cells/well) in medium containing 5 % FBS. When the cells reached 100 % confluence, the cells were incubated with the various Lf forms, each at a concentration of 5 mg/ml. After 24 h, the cells were washed twice with PBS and then were stained with reagents from an Annexin-V-FLUOS Staining Kit (Roche Diagnostics GmbH, Germany). Cell nuclei were counterstained with 5 µg/ml Hoechst 33342 (Life Technologies, USA). A fluorescence microscope, BX51 (Olympus Europe, Germany), with the appropriate filters was used to count the number of apoptotic cells (Annexin V-positive cells) and the number of necrotic cells (stained with propidium iodide) in five random fields of view. These numbers were compared with the total number of cells present (based on Hoechst staining) and the results are presented as percentages.

### Immunofluorescent staining of tight junctions

**Fig. 1:**
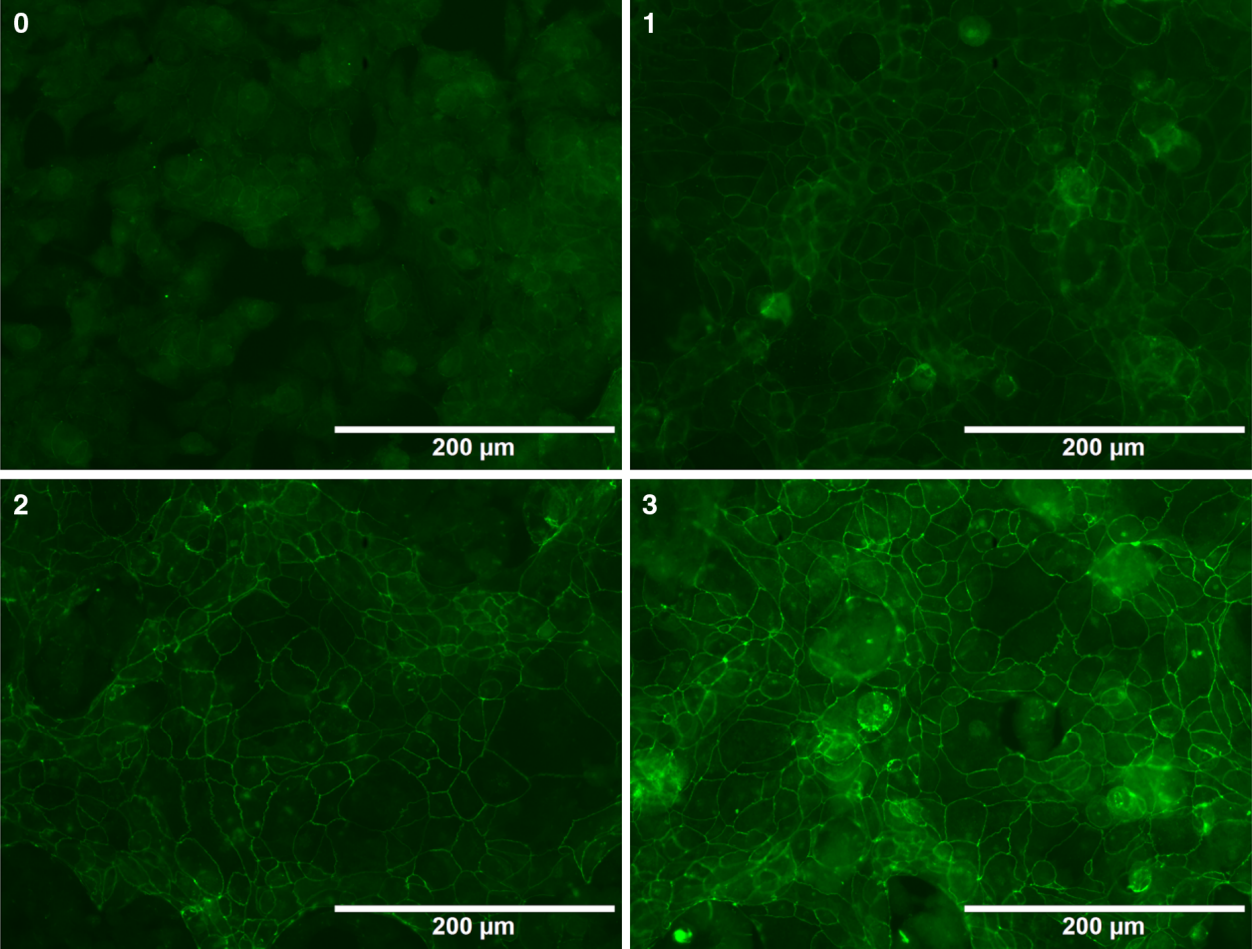
Fluorescent intensity corresponding to different levels of occludin expression in Caco-2 cells stained with a FITC-conjugated anti-occludin antibody. The semiquantitative scale used is represented in the images shown, with *0*–*3* indicating an absence, low levels, intermediate levels, or high levels of tight junction protein expression, respectively

Briefly, Caco-2 cells were seeded in 24-well plates (5 x 10^5^ cells/well) in medium containing 5 % FBS. After reaching 100 % confluence, the various forms of Lf (each 5 mg/ml) were added to the cells. After 24 h, the cells were washed twice with PBS and then were fixed for 5 min with methanol at 20 °C. After additional two washes with PBS, the cells were stained with a FITC-conjugated anti-occludin antibody (2.5 µg/ml, OC-3F10, Invitrogen, USA) overnight at 4 °C. A semiquantitative scale from 0 to 3 was used to indicate the absence, low levels, intermediate levels, and high levels of tight junction protein expression, respectively (Fig. 1).

### Cytokine production by resting and activated cells

I. The impact of the various Lf forms on secreted levels of pro- and anti-inflammatory cytokines in three in vitro cell culture models: Caco-2 cells (A), hMDMs (B), and J774A.1 cells (C).
  A. Caco-2 cells were plated as described in the apoptosis/necrosis assay. After reaching 100 % confluence, the various Lf forms were added to a final concentration of 5 mg/ml. After 24 h, supernatants were collected and assayed.
  B. Differentiated hMDMs after medium exchage into 0.5 % autological human serum medium were stimulated with the various Lf forms in a final concentration 5 mg/ml. Supernatants were collected 24 h later.
  C. J774A.1 cells were plated in 0.5 % FBS medium in 24-well plates (1 x 10^6^ cells/well). After the various Lf forms were added to a final concentration of 5 mg/ml for each, supernatants were collected 24 h later.
II. To evaluate the influence of the various Lf forms on the secretion of pro-inflammatory cytokines by immune cells activated by the presence of bacteria-derived molecules, two cell culture models were used: hMDMs and J774A.1 cells. Each set of cells was propagated and plated prior to these experiments as described above.
  A. The hMDMs were incubated with the various Lf forms at a concentration of 5 mg/ml. After 30 min, LPS (from *E. coli* O111:B4, Sigma-Aldrich) was added at a concentration of 10 ng/ml. The reverse sequence of additions (e.g., addition of LPS prior to addition of the various Lf forms) was performed as well. Supernatants were then collected 8 and 24 h later.
  B. J774A.1 cells were incubated with the various Lf forms at a concentration of 5 mg/ml. After 30 min, LPS (100 ng/ml) or an *E. coli* CM226 strain suspension was added. A suspension of thermally inactivated *E. coli* CM226 was used at a concentration corresponding to 10^7^ colony forming units per ml. This strain was isolated from the blood of VLBW newborns with clinical symptoms of sepsis. Prior to its use, the bacterial suspension in sterile phosphate-buffered saline was autoclaved at 121 °C and 1.02 bar pressure for 15 min. The reverse sequence of additions (e.g., addition of the bacteria-derived molecules prior to the various Lf forms) was performed as well. Supernatants were collected 24 h later.
  C. J774A.1 cells were propagated and incubated with the Lf forms (each at a concentration of 5 mg/ml) and LPS (100 ng/ml) as described in B. Minor modifications to this procedure included:
    1. LPS stimulation was maintained for 3 h prior to the addition of nLf.
    2. The cell culture medium was changed after the incubation with nLf and prior to the addition of LPS.

Cell supernatants were analyzed for levels of IL-1β, IL-6, IL-10, and TNF-α by using commercially available enzyme-linked immunosorbent assays (ELISAs) (BD Biosciences, San Jose, CA, USA) according to the manufacturer’s instructions.

### Statistical analysis

Statistical significance of differences between groups was analyzed using one-way analysis of variance (ANOVA) with post hoc Tukey’s multiple comparison test if significant differences were observed. A p value <0.05 was considered statistically significant. Analysis was performed using GraphPad Prism v. 5.01 (GraphPad Software, Inc., LaJolla, CA, USA).

## Results

### Effects of the various Lf forms on apoptosis and necrosis levels in Caco-2 cells

Lf enters the gastrointestinal passage during breastfeeding and undergoes partial degradation due to the acidic pH of the stomach (Fredrikzon and Hernell 1977; Hamosh et al. 1978; Roman et al. 2007). However, intact Lf has been detected in the stools of infants (Goldman et al. 1990). Therefore, we studied the interactions of various Lf species in a relevant model of human intestinal epithelium, the Caco-2 cell line. No statistically significant differences in the percentages of apoptotic and necrotic cells were detected following incubation of the Caco-2 cells with the various Lf forms tested (Table 2). Based on these results, it is predicted that none of the tested Lf forms should exert cytotoxic effects on human intestinal epithelial cells.

**Table 2:** Incubations of Caco-2 cells with the various Lf forms did not result in higher levels of apoptosis or necrosis

Sample	Living cells (%)	Apoptotic cells (%)	Necrotic cells (%)
DMEM - control	95.2 ± 0.3	0.12 ± 0.03	4.7 ± 0.2
apoLf (5 mg/ml)	94.6 ± 0.2	0.13 ± 0.02	5.3 ± 0.1
nLf (5 mg/ml)	94.6 ± 0.2	0.14 ± 0.02	5.3 ± 0.1
holoLf (5 mg/ml)	95.8 ± 0.2	0.10 ± 0.01	4.1 ± 0.1
MnLf (5 mg/ml)	96.2 ± 0.3	0.14 ± 0.02	3.7 ± 0.2

The values presented were calculated using data obtained from three independent experiments with each sample run in duplicate

### Effects of the various Lf forms on tight junction proteins

**Fig. 2:**
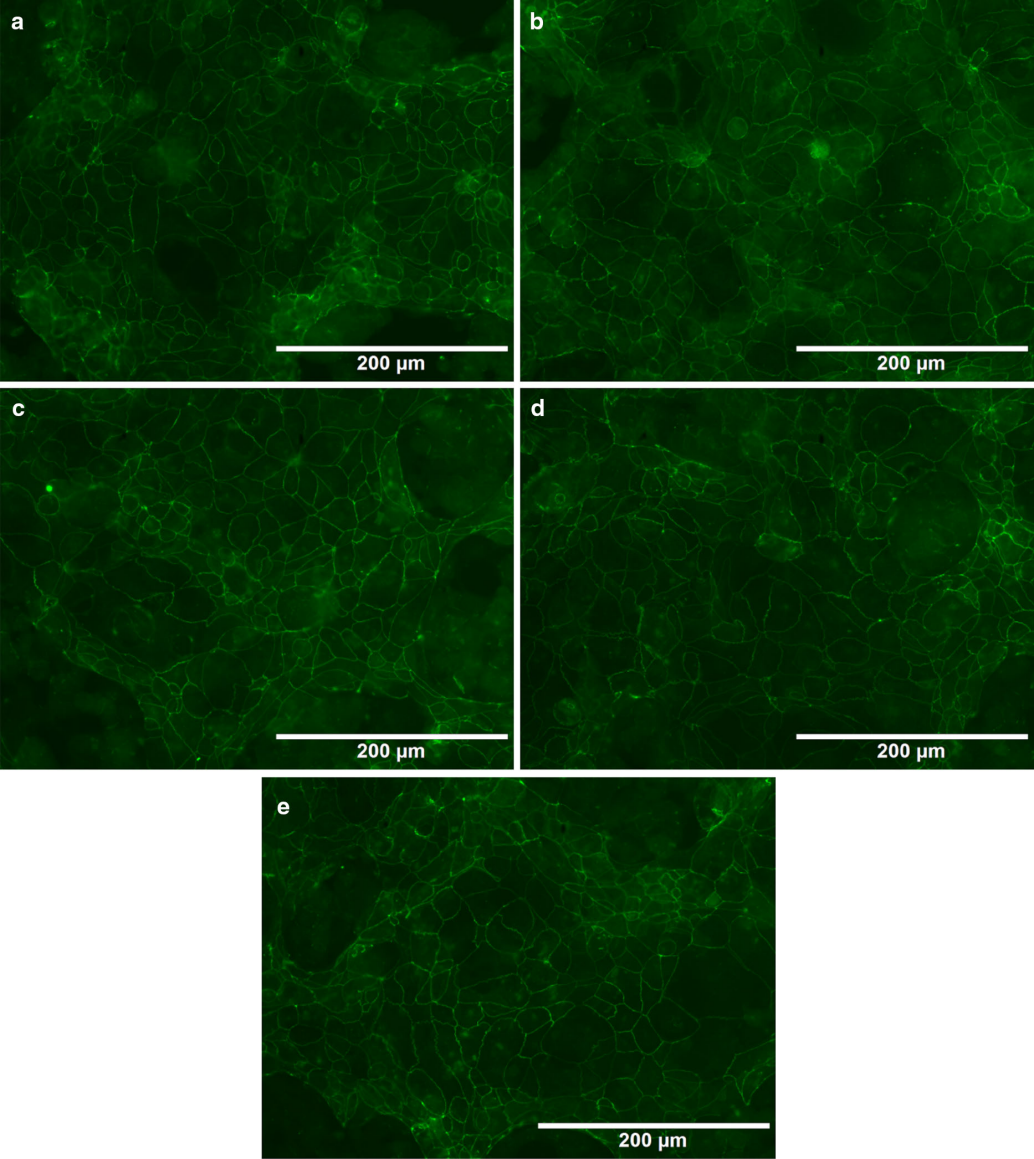
Lfs do not compromise the function of an epithelial barrier that is maintained by production of tight junction proteins. Immunofluorescent staining of occludin expression in Caco-2 cells is presented under stationary growth conditions (**a** and following incubation of the cells with apoLf, (**b)** nLf, (**c)** holoLf, (**d)** and MnLf, (**e)** with each Lf form present at a concentration of 5 mg/ml. The images are representative of three independent experiments that included duplicates of each sample

Integrity of the intestinal epithelium is determined by enterocyte cell viability and the presence of functional junctions between enterocyte cells. The latter are responsible for maintaining an efficient barrier between the intestinal lumen and surrounding tissues. Tight junctions are commonly composed of proteins such as claudin and occludin. When the Caco-2 cell line was incubated with the various Lf forms and then stained with an anti-occludin antibody, none of the tested Lf forms were found to affect the levels of occludin in the monolayer (Fig. 2). These results suggest that Lf does not enhance the formation of tight junctions, although it may preserve them.

### The various Lf forms tested do not affect cytokine secretion by resting cells

The secretion of several cytokines was detected after incubating Caco-2 cells and both human and murine macrophages (hMDMs and J774A.1 cells, respectively) with the various Lf forms. The levels detected for IL-1β, IL-6, TNF-α, and IL-10 were close to the detection limit of the ELISA kits used (data not shown). Therefore, we conclude that the Lf forms do not stimulate the secretion of the cytokines detected in either intestinal epithelial cells or macrophages.

### The various Lf forms inhibit secretion of pro-inflammatory cytokines by activated macrophages

**Fig. 3:**
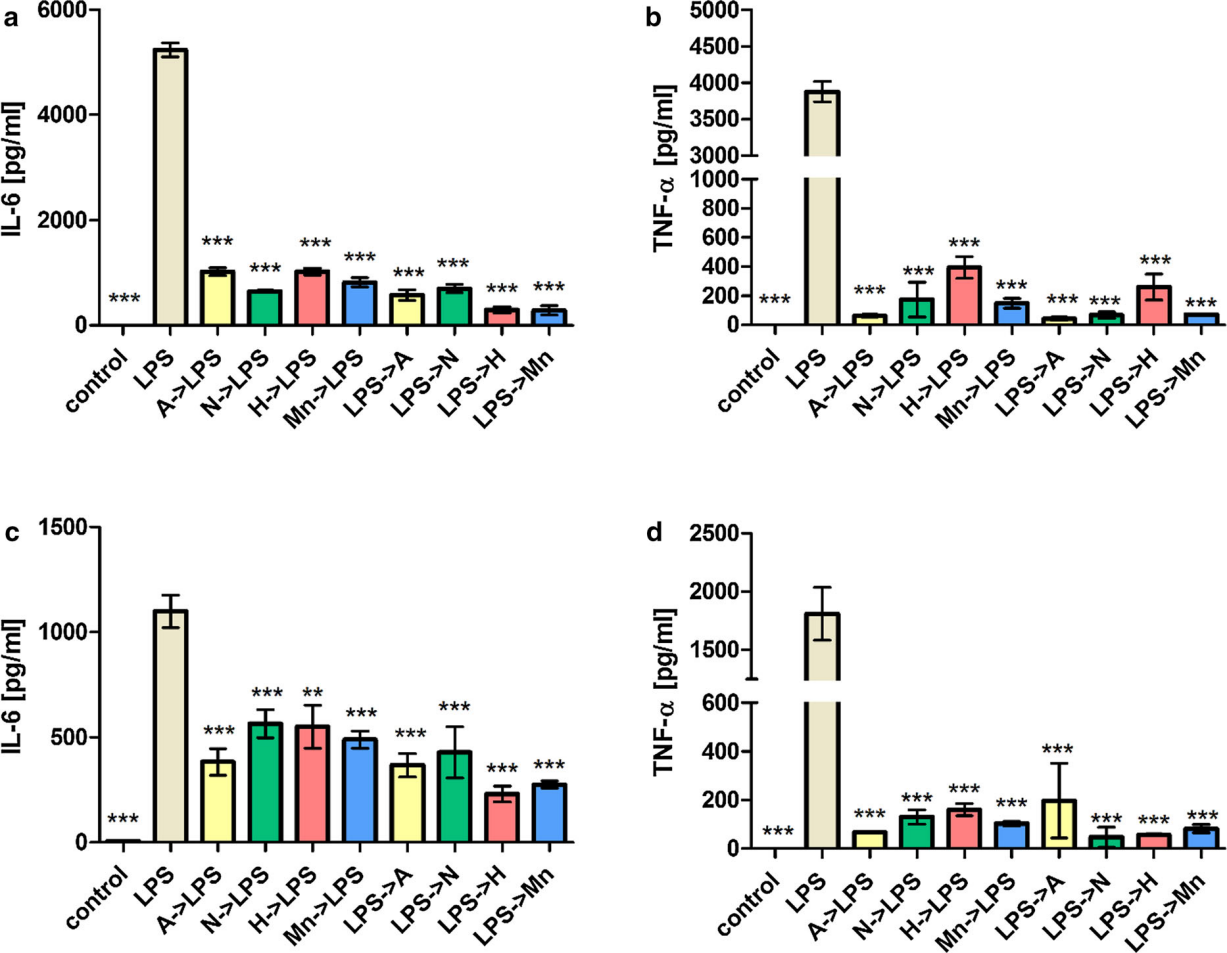
Secretion of pro-inflammatory cytokines induced by LPS stimulation of hMDMs was inhibited by the various Lf forms. IL-6 (**a**, **c**) and TNF-α (**b**, **d**) secretion after 8 h (**a**, **b**) and 24 h (**c**, **d**) in the presence of the various Lf forms: *A* apoLf, *N* nLf, *H* holoLf, *Mn* MnLf, or *LPS* lipopolysaccharide (100 ng/ml) are shown. Data in the graph represent the mean ± SEM from a representative experiment. The samples were analyzed in duplicate and were repeated with hMDMs obtained from two donors. Statistical significance was evaluated in comparison with hMDMs that were incubated only with LPS. *p < 0.05, ***p < 0.001

We also studied possible effects of the various Lf forms on LPS-dependent activation of human macrophages. In these experiments, secretion of IL-6 and TNF-α following LPS stimulation for 8 and 24 h were significantly inhibited in the presence of the various Lf forms (Fig. 3). The sequence of LPS and Lf additions also did not affect the degree of inhibition detected. There was no statistically significant difference between the inhibition achieved with the various Lf forms. However, we observed that increased iron saturation of Lf was found to correspond with a less pronounced inhibition of LPS stimulation in the case of detected TNF-α levels (Fig. 3b and d). In comparison with other Lf forms, apoLf exhibited the most potent inhibitory effect when it was added to the cell culture prior to LPS.

**Fig. 4:**
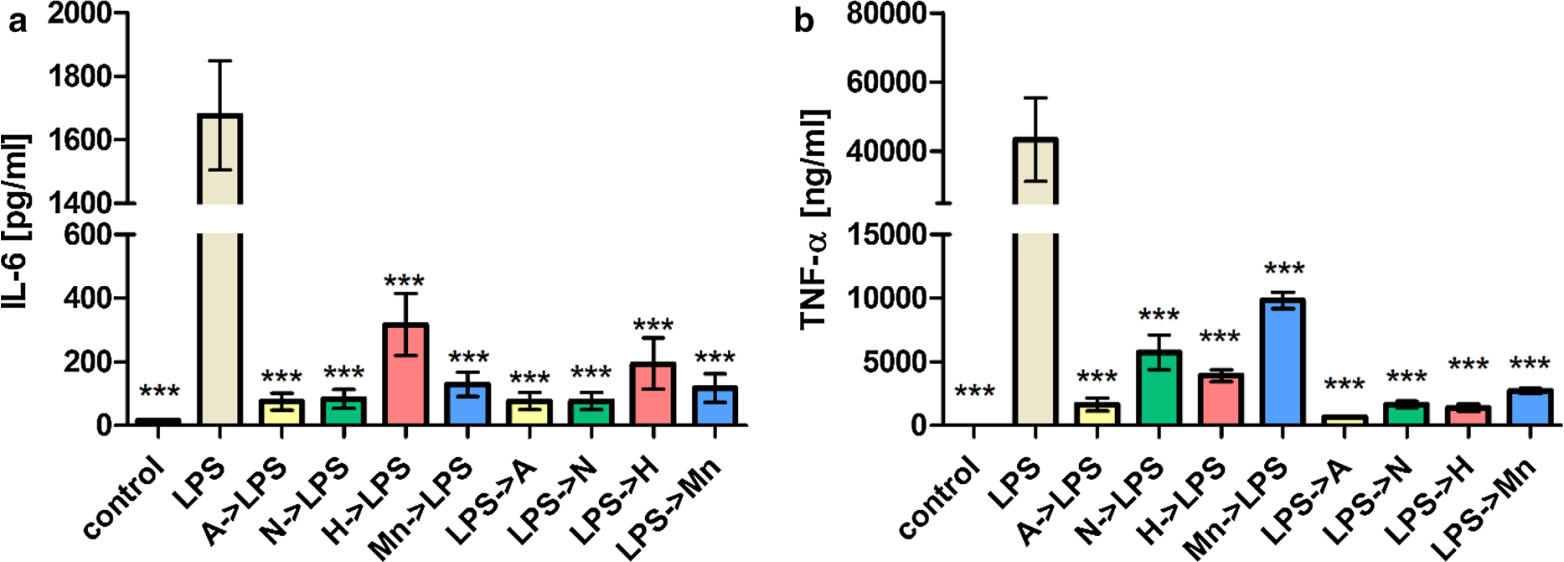
Lf forms inhibited the secretion of pro-inflammatory cytokines induced by LPS stimulation in J774A.1 cells. Secretion of IL-6 (**a**) and TNF-α (**b**) were detected 24 h after the addition of the various Lf forms to J774A.1 cells. *A* apoLf, *N* nLf, *H* holoLf,* Mn* MnLf, LPS (100 ng/ml). Data in the graph represent the mean ± SEM from two independent experiments that were performed in duplicate. Statistical significance was evaluated in comparison with J774A.1 cells that were only incubated with LPS. *p < 0.05, ***p < 0.001

To elucidate the mechanism(s) mediating the influence of the various Lf forms on cytokine secretion by activated immune cells, a well-established model employing the murine monocyte/macrophage J774A.1 cell line was used. The cells were incubated with the various Lf forms in combination with either LPS or thermally inactivated *E. coli* CM226. All of the tested Lf forms retained an ability to inhibit LPS-induced secretion of the pro-inflammatory cytokines, IL-6 and TNF-α (Fig. 4). Moreover, the observed differences were statistically significant for all of the Lf forms. However, the most potent inhibitory effect was observed for apoLf for both IL-6 and TNF-α.

**Fig. 5:**
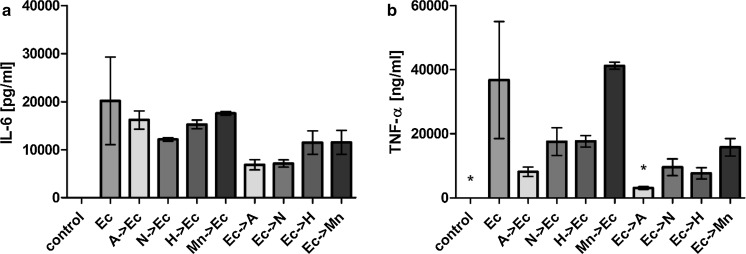
The impact of the various forms of Lf on the secretion of pro-inflammatory cytokines by J774A.1 cells in response to thermally inactivated *E. coli* CM226. Secretion of IL-6 (**a**) and TNF-α (**b**) were assayed 24 h after being cultured in the presence of:* A* apoLf,* N* nLf,* H *holoLf,* Mn* MnLf, or* Ec* a suspension of thermally inactivated *E. coli* CM226. Data in the graph represent the mean ± SEM from two independent experiments that included the samples run in duplicate. Statistical significance was evaluated in comparison with J774A.1 cells that were only incubated with *E. coli226*. *p < 0.05

When *E. coli* cells were used as a stimulating agent instead of LPS, the inhibitory effect on the secretion of pro-inflammatory cytokines by the various Lf forms was reduced (Fig. 5). Only a significant difference in the TNF-α levels that were detected after apoLf was added to the J774A.1 cells stimulated with *E. coli* CM226 was observed (Fig. 5b). However, in most cases, only minor inhibition of IL-6 and TNF-α secretion were detected. Again, for TNF-α levels, we found apoLf to be the most potent form to inhibit the pro-inflammatory response. These results support the importance of Lf interactions with LPS, and they also suggest that the anti-inflammatory activity of Lf is not as potent when a more complex mixture of bacterial antigens is used for the stimulation of immune cells.

### Lf–LPS interactions are crucial for the anti-inflammatory activity of Lf

**Fig. 6:**
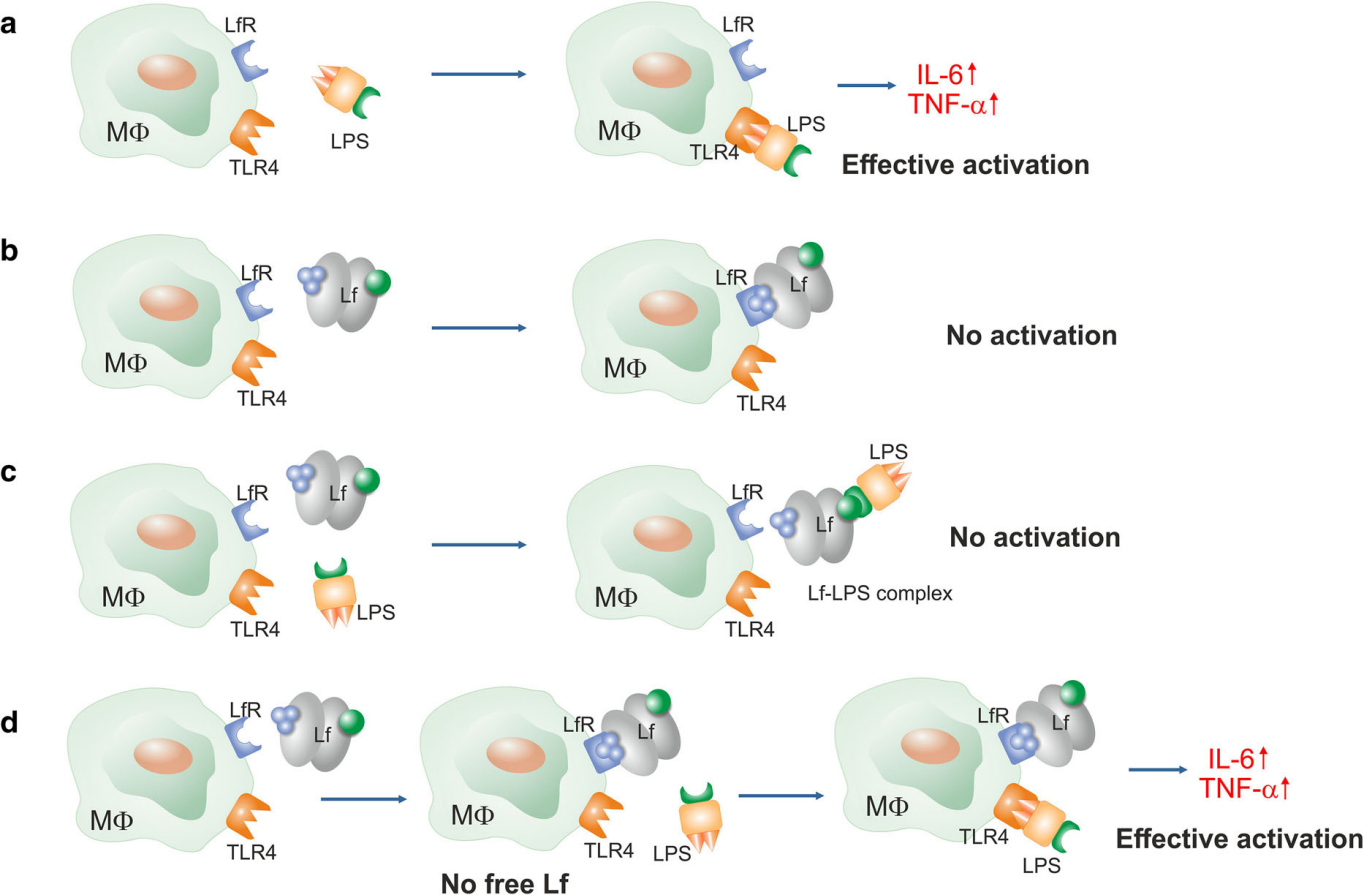
Interactions between Lf and LPS in the presence of macrophage. **a** Activation of macrophage with LPS via TLR4 induces the secretion of pro-inflammatory cytokines (IL-6, TNF-α). **b** Lf itself does not induce macrophage activation. **c**. A mixture of Lf and LPS does not activate macrophage due to the formation of a Lf–LPS complex that is unable to activate TLR4. **d** Free, non-bound Lf is needed to prevent LPS-induced macrophage activation. Removal of free Lf by a medium change performed prior to the addition of LPS resulted in an effective activation of macrophage.* MΦ* macrophage cell,* Lf* lactoferrin,* LPS* lipopolysaccharide,* LfR* lactoferrin receptor,* TLR4* toll-like receptor 4

To elucidate whether the secretion of cytokines by activated immune cells is dependent on the binding of Lf to its cognate receptor on macrophages or the direct binding and neutralization of LPS, we introduced several modifications to the experimental model described above. Instead of adding only a single agent (Lf or LPS) or two agents sequentially within a short time frame (Fig. 6a–c), we delayed the addition of Lf to the LPS-stimulated cells from 30 min to 3 h. The levels of secreted IL-6 and TNF-α following LPS treatment versus *E. coli* CM226 treatment were 3 versus 27 % and 11 versus 55 %, respectively in each case. Thus, Lf-dependent inhibition of cytokine production was reduced (Table 3).

**Table 3:** The effect of native lactoferrin (N) on LPS-induced secretion of IL-6 and TNF-α by J774A.1 cells

Sample	IL-6		TNF-α	
	Concentration (pg/ml)	Maximal response (%)	Concentration (pg/ml)	Maximal response (%)
LPS	22,280 ± 5175	100	20,536 ± 2354	100
N + LPS	700 ± 100	2.8 ± 0.4	4873 ± 1199	23.7 ± 5.8
LPS + N	785 ± 35	3.1 ± 0.1	2260 ± 381	11.0 ± 1.8
LPS (3 h) + N	6685 ± 1180	26.6 ± 4.7	11,350 ± 537	55.2 ± 2.6
N- > LPS*	21,086 ± 1766	83.81 ± 7.0	10,640 ± 714	51.8 ± 3.5

The results are expressed as a percentage of maximal response (mean ± SEM from triplicates assayed in one of two independent experiments)

N + LPS denotes that LPS was added 30 min after N was added to the cells

LPS + N denotes that LPS was added 30 min prior to N addition

LPS(3 h) + N denotes that incubation with LPS was extended to a period of 3 h

N- > LPS* denotes that the medium was changed after the nLf incubation and LPS was added to nLf-free medium

Furthermore, when the cell culture medium was changed after the nLf incubation, LPS-induced secretion of proinflammatory cytokines was only partially inhibited by Lf. For example, the maximal response percentage increased from 3 to 83 % for IL-6 and from 24 to 52 % for TNF-α (Fig. 6d; Table 3). These results confirm that binding of Lf by macrophage does not affect the secretion of cytokines IL-6 and TNF-α following stimulation by LPS. Unbound, free Lf neutralizes LPS and prevents its binding to receptors on macrophage.

## Discussion

Bovine lactoferrin, when taken orally, is considered safe and it has no known detrimental effects on human health. Thus, for decades, bovine Lf has been used as a diet supplement and an additive to infant formula. In the present study, the effects of a high concentration of Lf (5 mg/ml) were investigated. This concentration corresponds to the levels of Lf that are naturally occurring in human colostrum, the milk that infants should receive in the first hours of life. Based on the high concentration of Lf that is present in human colostrum and milk, it is hypothesized that this protein plays a crucial role in maintaining homeostasis in the neonatal gut niche. Such high levels of Lf are also relevant for diet supplements for neonates, and Lf supplementation has proven effective in preventing neonatal sepsis (Manzoni et al. 2009, 2012).

It is commonly accepted that Lf is an important component of innate immunity and is capable of interacting with microorganisms (via an iron sequestration mechanism) and microbial molecules (by neutralization of LPS) that are present in the intestinal lumen. However, Lf may also affect host cells—including enterocytes and immune cells—that possess cognate receptors for Lf. For example, Lf has been shown to affect the proliferation of enterocytes and their differentiation, and also has a significant impact on macrophage function via the production of cytokines and inflammatory intermediates.

Despite a large body of evidence that supports the beneficial impact of various Lf preparations on human health, there is still very little information regarding the mode of action of Lf and whether saturation of the protein with Fe and Mn affects its function. An aim of the present study was to elucidate the impact of metal (Fe, Mn) saturation levels on bovine Lf in regard to its function in maintaining the gut epithelial barrier that consists of intestinal epithelial cells, as well as immune cells of the MALT system.

The present data confirm that Lf does not mediate an adverse effect on the barrier formed by intestinal epithelium cells. At a concentration of 5 mg/ml, both native Lf and the various forms of Lf that differed in their metal saturation levels (e.g., apoLf, holoLf, and MnLf) did not induce a cytotoxic effect on the Caco-2 cell line as measured in the apoptosis/necrosis assays that were performed. The present results are also consistent with the non-toxic profile of Lf obtained from various cells lines at similar concentrations (Ajello et al. 2002; Fillebeen et al. 1999).

Preservation of a functional intestinal barrier relies heavily upon the formation of specialized intercellular junctions. Tight junction proteins, such as claudin 1 and occludin, form macromolecular complexes that make the enterocyte monolayer impermeable to substances present in the intestinal lumen. Our results suggest that neither native Lf, nor the forms of Lf that differed in metal saturation, affected the levels of occludin in the Caco-2 monolayer. We observed no change in the production of tight junction proteins upon incubation with various lactoferrin forms—however, the stationary level of those junctions was preserved to the best of our knowledge, these are the first data regarding the potential influence of Lf on the presence of occludin in a monolayer. However, we did not confirm the previous observation that Lf can decrease the permeability of Caco-2 cells to polysaccharide molecules (Hirotani et al. 2008) in our model.

When Caco-2 cells were incubated with the various Lf forms tested, levels of pro- (IL-1β, IL-6, TNF-α) and anti-inflammatory (IL-10) cytokines were not detectable. These results indicate that Lf itself, without a distinction between various metal saturation levels, does not induce an inflammatory response in enterocytes. Such results are in concord with the literature data based on studies using lower concentrations of Lf (Berlutti et al. 2006) and confirm that bovine Lf preparations with different metal saturation levels do not alter the function of an epithelial cell monolayer.

Since our results clearly suggest that none of the tested forms of Lf affect the intestinal barrier, it was subsequently investigated whether the various Lf forms interact similarly with LPS and *E. coli*, which are representative components of gut microbiota. We also investigated how these interactions might affect cytokine production by inflammatory cells, particularly macrophages).

The various Lf forms did not induce the secretion of IL-6 or TNF-α by resting human monocyte-derived macrophage. However, the various Lf forms inhibited LPS-induced secretion of both of these pro-inflammatory cytokines by hMDMs 8 and 24 h after the addition of the Lf forms. This same effect was observed when the various Lf forms were added to the cell culture medium 30 min before or after LPS incubation.

Similar results were obtained in our experiments with a well-described murine macrophage model, J774A.1 cells. Cytokine secretion by these macrophages following LPS activation was inhibited when the various Lf forms were added prior to, or after, the addition of LPS to the cell culture medium. These results indicate that neither binding of LPS, nor neutralization of LPS, are significantly affected by the metal saturation status of Lf. However, the strongest inhibitory effects were associated with the iron-depleted form of Lf, apoLf, which—to our best knowledge—has never been shown before. Furthermore, by changing the medium after the nLf stimulation step, we demonstrate that free Lf present in molar excess is necessary for LPS neutralization.

The results of the present study are in agreement with the results of other reports on the immunoregulatory properties of Lf. It has been determined that Lf binds lipid A with high affinity, thereby neutralizing LPS since lipid A is not further recognized by LBP or by the receptors present on immune cells such as TLR4 (Jiang and Lönnerdal 2012). At lower concentrations (e.g., 0.01–1 µg/ml), human Lf has been shown to inhibit IL-6 secretion by LPS-activated THP-1 cells (Mattsby-Baltzer et al. 1996). Similarly, bovine Lf (0.1–1.0 mg/ml) has been shown to reduce the secretion of TNF-α by LPS-activated Raw264.7 macrophage and THP-1 cells (Choe and Lee 2000). Moreover, both human (0.2 mg/ml) and bovine (0.5 mg/ml) Lf have effectively inhibited the secretion of several pro-inflammatory cytokines (e.g., TNF-α, IL-1β, and IL-8) in THP-1 macrophages that were challenged with LPS (Håversen et al. 2002). A more recent report demonstrated that pre-treatment, yet not co-treatment, with bovine Lf (0.125–2 mg/ml) diminished LPS-induced TNF-α secretion by THP-1 cells (Tian et al. 2010). Thus, while the results of the present study are based on experiments that used Lf at a high concentration (5 mg/ml), these results are in accordance with previous results that were obtained using lower concentrations of Lf. A higher concentration of Lf would also be predicted to be more potent in neutralizing LPS (compared to the conditions described in the studies above). The inhibitory effect of Lf on IL-6 and TNF-α secretion was observed in the present study when Lf was added before or after the addition of LPS. It is also worth noting that the impact of Lf’s metal saturation state on the ability of Lf to neutralize LPS has not previously been elucidated.

Several reports indicate the potential for Lf to mediate pro-inflammatory effects on immune cells. For example, bovine Lf at 0.1 mg/ml was shown to induce the secretion of TNF-α, IL-8, and nitric oxide (NO) from rat bone marrow-derived macrophages (Sorimachi et al. 1997). Later reports provide results that support activation of the NF-κB pathway by human Lf (0.5 mg/ml) in THP-1 cells (Ando et al. 2010). Our results are distinct from these studies, and this may be due to differences in the concentrations of Lf that were used. In the present study, an Lf concentration based on maternal colostrum and milk levels relevant to neonate feeding in the first days of life was used.

Using murine macrophages we confirmed that the clear inhibitory effects on pro-inflammatory cytokine secretion that were observed for LPS-activated macrophage in the presence of the various Lf forms tested were not as potent when the immune cells used were activated by a more complex mixture of molecules derived from thermally inactivated *E. coli*. These results further confirm the importance of Lf interactions with specific molecules—namely LPS. To the best of our knowledge, this is the first report of a comparison of Lf potential to inhibit cytokine production by immune cells that employed both LPS and a mixture of native bacteria-derived molecules.

Another important function of Lf in the intestinal lumen and at sites of inflammation is its interaction with cognate receptors on host cells. Lf can be bound and internalized via specific LfRs that are present on enterocytes and immune cells. However, there have been several reports that Lf can stimulate immune cells via TLRs as well (Ando et al. 2010). The present results do not confirm the ability of Lf to affect TLRs. The addition of Lf to macrophage at high concentrations is predicted to achieve a saturation of LfRs that are present on macrophages. In the present study, the use of Lf at a high concentration did not affect LPS-induced responses when free, non-bound Lf was removed from the culture medium. Therefore, our results suggest that free Lf is necessary for binding and neutralizing LPS.

**Fig. 7:**
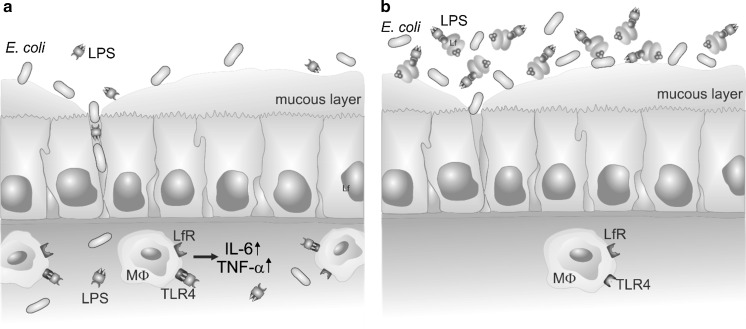
Potential impact of Lf on homeostasis of the intestinal barrier in neonates. **a**. In the absence of Lf, disruption of the mucous layer results in a loss of intestinal integrity and the translocation of microorganisms and microbial-derived antigens from the intestinal lumen to peripheral tissues. Infiltrating immune cells also initiate an acute pro-inflammatory response that is mediated by cytokines such as IL-6 and TNF-α. **b**. Diet supplementation with Lf leads to neutralization of microbial antigens (namely LPS) in the intestinal lumen and a reduced inflammatory response.* MΦ* macrophage,* Lf *lactoferrin,* LPS *lipopolysaccharide,* LfR* lactoferrin receptor,* TLR4* toll-like receptor 4

The results of the present study suggest that oral supplementation with Lf may have a beneficial effect on the homeostasis of the neonatal gut niche (Fig. 7). We have shown that Lf is able to neutralize molecules derived from Gram-negative bacteria—mostly LPS—and this capacity does not depend on the metal saturation status of Lf. Furthermore, we have demonstrated that free Lf present in molar excess (e.g., not bound to its cognate receptor) is crucial for binding and neutralizing LPS, as well as preventing interactions between Lf and components of the immune system. The present data also show that even high concentrations of Lf do not have a negative influence on the barrier established by tight junctions within a Caco-2 monolayer. High doses of Lf are desirable since they allow LfRs on enterocytes to be saturated and the excess Lf can neutralize LPS. Our studies did not reveal significant differences in the anti-inflammatory properties of the various Lf forms—however, the most potent inhibitory effect on the LPS-dependent activation of the macrophages was noted for apolatoferrin. It has previously been demonstrated that native Lf can inhibit the growth of some pathogenic species. It is predicted that the apoLf form would also be able to sequester iron and inhibit the growth of pathogenic bacteria. However, we believe that manganese-saturated Lf would have a better impact on gut homeostasis due to its observed prebiotic properties towards *Lactobacillus* strains. Thus, while the release of manganese ions from MnLf has the potential to enhance *Lactobacillus* species growth in the intestinal lumen, apoLf could bind excess iron present in the gut niche.
